# Traditional Nets Interfere with the Uptake of Long-Lasting Insecticidal Nets in the Peruvian Amazon: The Relevance of Net Preference for Achieving High Coverage and Use

**DOI:** 10.1371/journal.pone.0050294

**Published:** 2013-01-02

**Authors:** Koen Peeters Grietens, Joan Muela Ribera, Veronica Soto, Alex Tenorio, Sarah Hoibak, Angel Rosas Aguirre, Elizabeth Toomer, Hugo Rodriguez, Alejandro Llanos Cuentas, Umberto D'Alessandro, Dionicia Gamboa, Annette Erhart

**Affiliations:** 1 Prince Leopold Institute of Tropical Medicine, Antwerp, Belgium; 2 Partners for Applied Social Sciences, PASS International, Tessenderlo, Belgium; 3 Instituto de Medicina Tropical Alexander von Humboldt, Universidad Peruana Cayetano Heredia, Lima, Peru; 4 Organismo Andino de Salud-Convenio Hipólito Unanue, Proyecto PAMAFRO, Lima, Peru; 5 The MENTOR Initiative, Crawley, United Kingdom; 6 Medical Research Council Unit, Fajara, The Gambia; 7 Departamento de Ciencias Celulares y Moleculares, Facultad de Ciencias y Filosofía, Universidad Peruana Cayetano Heredia, Lima, Peru; Kenya Medical Research Institute (KEMRI), Kenya

## Abstract

**Background:**

While coverage of long-lasting insecticide-treated nets (LLIN) has steadily increased, a growing number of studies report gaps between net ownership and use. We conducted a mixed-methods social science study assessing the importance of net preference and use after Olyset® LLINs were distributed through a mass campaign in rural communities surrounding Iquitos, the capital city of the Amazonian region of Peru.

**Methods:**

The study was conducted in the catchment area of the Paujil and Cahuide Health Centres (San Juan district) between July 2007 and November 2008. During a first qualitative phase, participant observation and in-depth interviews collected information on key determinants for net preference and use. In a second quantitative phase, a survey among recently confirmed malaria patients evaluated the acceptability and use of both LLINs and traditional nets, and a case control study assessed the association between net preference/use and housing structure (open *vs.* closed houses).

**Results:**

A total of 10 communities were selected for the anthropological fieldwork and 228 households participated in the quantitative studies. In the study area, bed nets are considered part of the housing structure and are therefore required to fulfil specific architectural and social functions, such as providing privacy and shelter, which the newly distributed Olyset® LLINs ultimately did not. The LLINs' failure to meet these criteria could mainly be attributed to their large mesh size, transparency and perceived ineffectiveness to protect against mosquitoes and other insects, resulting in 63.3% of households not using any of the distributed LLINs. Notably, LLIN usage was significantly lower in houses with no interior or exterior walls (35.2%) than in those with walls (73.8%) (OR = 5.2, 95CI [2.2; 12.3], p<0.001).

**Conclusion:**

Net preference can interfere with optimal LLIN use. In order to improve the number of effective days of LLIN protection per dollar spent, appropriate quantitative and qualitative methods for collecting information on net preference should be developed before any LLIN procurement decision is made.

## Introduction

The use of malaria preventive measures such as insecticide treated nets (ITNs) and Long-Lasting Insecticidal Nets (LLINs), by populations living in malaria endemic countries is a key aspect of the Global Malaria Action Plan's established goals for 2015 [1] and is paramount to achieve the targets set by the World Health Organization (WHO) and the Roll Back Malaria Partnership [1]–[2]. The impact of ITN use on reducing malaria mortality and morbidity has been repeatedly shown [3]–[5] and has lead to a steady increase of LLIN coverage in the last decade via mass distribution campaigns and keep-up strategies [1]–[2]. As coverage increases, establishing the actual *use* of available LLINs is becoming the next hurdle as studies increasingly report a gap between net ownership and use [6]–[10] that is affected by factors that are still poorly defined [11].

User preference for certain net characteristics, e.g. mesh size, colour, dimensions and fabric, may have a direct impact on net use. In terms of malaria prevention, the problem arises when users prefer locally produced or bought bed nets that are untreated with insecticide as they offer less protection against mosquito bites. In addition, their use could interfere with the uptake of LLINs distributed by Malaria Control Programmes. Under these conditions, the LLINs could either be sold, stored for later or reserved for alternative uses (e.g. fishing, crop protection [12]) resulting in wasted donor resources and potential environmental hazards [13].

Currently, the World Health Organization Pesticide Evaluation Scheme (WHOPES) has approved several LLIN-brands, each with different design characteristics (shape, material, mesh size, insecticide type and insecticide application) [14]. However, due to the lack of robust data on the impact of user preferences on LLIN use and the consequent lack of information for decision-making, net preference is currently not included in the procurement process [15].

For several donors, including the Global Fund for Aids, Tuberculosis and Malaria (GFATM), current procurement practices do not allow the selection of a specific LLIN brand. Specifying the size, shape and colour can be included in the tender process as far as it does not impact on the cost [16]. The type of material (polyester, polyethylene, polypropylene), denier or mesh size are brand specific and cannot be indicated on the tender as by doing so one or several brands of LLIN may be excluded from the competitive procurement process. In addition, other financial and legal factors such as registration in the recipient country, the lowest evaluated bid (including price, delivery cost and schedule), stock availability, delivery deadlines and a transparent and open tender process [17]–[18] influence the type of net procured.

One of the currently debated questions is how users' preferences influence net use. Anecdotal observations done by the authors in several countries (Vietnam, Cambodia, Madagascar, Kenya, Tanzania, Peru) suggest that LLIN-use can be limited by a preference for locally bought, untreated nets, as they offer certain options (different colours, shapes, mesh size, opening) that distributed LLINs do not. Furthermore, modification of existing nets to suit users' personal preferences, as reported in Senegal [12] and Kenya (Sarah Hoibak, personal communication) where local tailors convert rectangular LLINs into a conical shape, are an additional indication of the potential relevance of net preference for LLIN use. We therefore explored the importance of users' net preferences for bed net use in the Peruvian Amazon where use is reported to be exceptionally high, reaching almost 100% [19]–[22]. The most commonly used net is the traditional *tocuyo* net produced from a white opaque, muslin-like, cotton fabric that can be locally produced or purchased [21], [22] but that is not treated with insecticide. The challenge for the Peruvian Malaria Control Program in this setting has therefore not been the promotion of bed net use itself but rather the replacement of commonly used untreated nets with ITNs or LLINs. A first attempt to do so was carried out in 1997. In response to a malaria outbreak, the Ministry of Health (MoH) distributed 82,000 conventional ITNs to replace the traditional nets [22]. A decade later, in 2007, the PAMAFRO project [23] coordinated the distribution of LLINs (Olyset® net, Sumitomo Chemicals, Japan) in all endemic districts of the Department of Loreto [24]. Between 2007 and 2010, a total of 242,312 LLINs were distributed, each LLIN covering on average 2.7 people [25]. Though apparently ideal to replace the *tocuyo*-nets, no data were collected on whether these LLINs would actually meet the needs and expectations of the local population. For this reason, we carried out a mixed methods social science study to determine user preference, acceptability and use of the distributed LLINs. The results presented are part of a larger social science study on vulnerability to malaria in the Peruvian Amazon, a part of which, focusing on *P.vivax* treatment adherence, was published elsewhere [26]. The study was ancillary to a larger on-going cohort study on *P.vivax* morbidity [27], carried out in the framework of the Institutional Collaboration between the Institute of Tropical Medicine (ITM), Antwerp-Belgium and the ITM Alexander von Humboldt (ITM-AvH), Universidad Peruana Cayetano Heredia, Lima-Peru.

## Methods

### Study site and population

The study was conducted in the catchment area of the Paujil and Cahuide Health Centres in San Juan district (Loreto Department, Peruvian Amazon) situated along the road connecting Iquitos City to the port city of Nauta [26]. Malaria distribution in Loreto is highly heterogeneous, containing foci of high incidence, such as the district of San Juan, which in 2007 reported the highest number of cases in the region (total 4,075 for a population of 108,353) [28]. Malaria transmission is perennial with a peak during the rainy season (from November to May), and the majority of malaria cases are due to *Plasmodium vivax.* All age groups are at risk for malaria infection, though adults more than children [29], [30]. The main vector is *Anopheles darlingi*, a highly anthropophilic species [31], [32].

In 2007, a total of 15,207 Olyset® LLINs were distributed to 5,069 families in 55 localities of San Juan district, which also includes our study area [33]. Communities in the study region are located near the Itaya and Nanay Rivers and within 15 km from the nearest health centre [30]. Despite the road, accessibility during the rainy season is difficult for those communities that can only be reached by boat. Local subsistence strategies include slash and burn agriculture, fishing, hunting, and small-scale coal production. People occasionally engage in fish farming, logging, small commercial activities and salaried employment as grounds' keepers or cultivators for institutions, farms and enterprises belonging to wealthier Iquitos' residents. The population in the catchment area of Paujil and Cahuide was estimated in 2007 at 5,239 inhabitants and consisted mainly of *mestizos* -referring to all Peruvians that cannot be clearly identified as belonging to any ethnic minority population.

### Research strategy

The research strategy consisted of triangulating different methodologies and data collection techniques. The chosen mixed methods design consisted of an introductory exploratory qualitative strand, followed by in-depth qualitative and quantitative strands (represented in standard mixed methods notation as [qual→qual+quan] [34]). During a first exploratory strand, qualitative ethnographic data were collected in local communities to acquire an in-depth understanding of the study setting and population in terms of the research question. In a second phase, concomitantly to the on-going qualitative data collection, two subgroups/settings were identified as most relevant for quantitative studies. The first group consisted of symptomatic malaria patients identified at the local health centre as they represented a confirmed high-risk group for malaria. The second setting consisted of households living in open houses, which exemplified the social use of sleeping space in the study area and its importance for net use and preference. The sampling frame of villages, individuals and households for both qualitative and quantitative data collection were selected from the same study area.

### Qualitative strand

An ethnographic study was carried out during two field stays of approximately three months each (Jul–Oct 2007; Aug–Nov 2008) representing an initial exploratory phase and a second triangulating phase parallel to the quantitative data collection. Data collection techniques consisted of participant observation and interviews. Participant observation was essential to assess which factors were relevant for net preference and use. Through the observation of daily activities, factors relevant to net use were recorded. This further allowed carrying out reiterated informal conversations and interviews to build up informant confidence during the interviews. Since the research question was related to the evaluation of the uptake of a public health intervention - free-of-charge LLIN-distribution- participant observation was a key aspect of the methodology as it is a respondent independent technique used to overcome the bias often inherent to self-reporting techniques such as standardized questionnaires. *Interviewing*: Interviews were carried out after respondents' verbal consent and, when applicable, were recorded and transcribed. In these cases when the interviewer(s) considered recording or note taking inappropriate in front of the respondent the conversation was written down immediately after the interview.

#### Sampling

10 villages were theoretically selected on the basis of malaria endemicity, accessibility, economic activities and emergent findings. Fieldwork was carried out in the communities of El Dorado, 13 de Febrero, Nuevo Horizonte, Ex-Petroleros, 12 de Abril, Cahuide, Villa Buen Pastor, Paujil, 24 de Junio and El Triunfo. Sampling for all informal and formal interviews was purposive. Informants were gradually selected according to relevant variables such as gender, age, subsistence strategy, locality, LLIN-use, and housing structure to allow for maximum variation in the sample.

### Quantitative strand

#### 1. Malaria Patients Survey

A closed ended questionnaire on net preference, LLIN acceptability and bed net use was administered one year after their distribution to a random sample of 158 individuals selected from the Cahuide and Paujil Health Centre clinical database that included all malaria patients identified between January 2005 and July 2007 (total = 1,072). The survey focused on recently identified malaria patients (confirmed by microscopic examination at the health centre), and aimed at collecting basic data on the perceived benefits and use of LLINs and traditional nets among this high-risk group. The initial sample size calculation estimated that a minimum sample of 160 individuals was required to estimate at least 60% LLINs use among malaria patients, with a 7% precision and 95% confidence level. A standardized pre-coded questionnaire was administered to all participants by the study team; for children <18 years old, the questionnaire was administered to parents/guardians (for more details see [26]). The survey examined the perceived benefits, inconveniences and risks of LLIN use in comparison to traditional nets, including the adequacy of LLINs for the local context in terms of climatic factors (heat/cold), local beliefs, and insect abundance.

#### 2. Housing study

**Figure 1:**
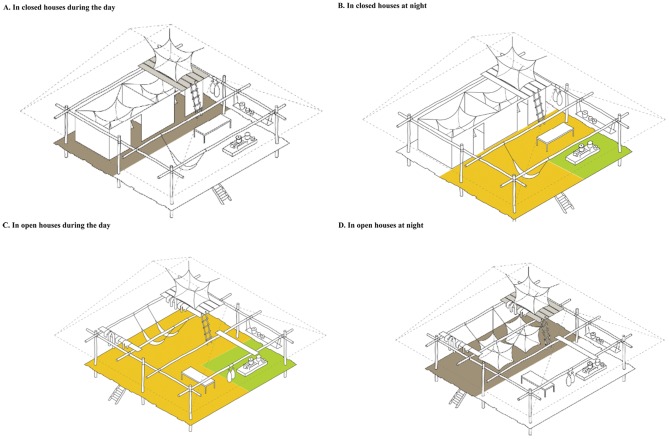
Social use of space in open versus closed houses.

The second quantitative study investigated aspects of net preference and use related to the structure of local housing and the social and cultural use of housing space, following information collected during the initial qualitative research phase. A case-control design was preferred for the comparison of families living in *open* houses to their geographically closest neighbours living in *closed* houses, the former being chosen exhaustively from the MoH housing structure inventory. The MoH inventory is an official register that identifies and categorizes all houses prior each indoor residual spraying campaign. A total of 35 *open* houses could be identified in the study area at the time of the survey. Remaining *open* houses could either not be located, their households had moved out of the study region or the structure of the house had been modified since the last MoH inventory. *Case definitions*: (1) *Open* houses were operationally defined as houses without inner and outer walls (Figure 1, A&B). *Closed* houses were defined as structures with at least four walls (Figure 1, C&D). All houses that did not correspond to the above-defined criteria, i.e. rooms made of canvas, houses with only one outside wall and no inner walls, or one wall at the back of the house, were excluded. The Housing Study included questions regarding both the primary intended function of LLINs (protection against malaria) and secondary benefits (privacy, protecting from cold, insects, etc.) of bed net use.

### Data analysis

#### Qualitative Data

Qualitative data collection and analysis were performed concurrently and data analysis was an iterative process. Preliminary data were intermittently analysed in the field, and preliminary results were then translated into the question guides for follow-up interviews. Continuous validity checks were used to confirm or refute initial results until saturation was reached and the data could be theoretically supported. Analytic induction involved the iterative testing of theoretical ideas, which was used to refine and categorize themes grounded in the data while emerging themes were evaluated in dialogue with existing social science theory. This resulted in an analytical framework that was then systematically applied in the data analysis. Data were entered, managed and analysed in NVivo 8 Qualitative Data Analysis software (QSR International Pty Ltd. Cardigan UK).

#### Quantitative data

All survey data were entered and cleaned in Epi Info 6.04 (CDC, Atlanta; WHO Geneva, 1996) and analysed in SPSS (PASW Statistics 18, SPSS Inc, IBM, Chicago 2010). For the Malaria Patients Survey, descriptive statistics were computed using proportions with corresponding 95% confidence intervals. The main outcome variables were net preference and net use according to various perceived primary and secondary benefits, climatic conditions and housing structure. For the case-control study, crude odds ratios were computed with corresponding Fisher Exact p-values, to assess the odds of net use in *open* and *closed* houses.

### Ethical clearance

The study was approved by the Ethical Committee of the University of Antwerp, Belgium, and the Ethical Review Committee of the Universidad Peruana Cayetano Heredia, Lima, Peru. For qualitative data collection, interviewers followed the guidelines of the Code of Ethics of the American Anthropological Association (AAA) [35]. As proposed by the AAA, all interviewees were informed before the start of the interview about project goals, the topic and type of questions, their right to refuse being interviewed, to interrupt the conversation at any time, to withdraw any given information during or after the interview, and about the intended use of the results for scientific publications and reports to health authorities. Oral consent was preferred for all data collection since the interviewees were not put at any risk of being harmed physically or psychologically. The oral consent procedure was approved by the above-mentioned Ethical Review Committees. We expected that the act of signing one's name when providing information during interviews could be a potential reason for mistrust [36] and could lead to increased social desirability bias and therefore less reliable data.

## Results

### LLIN Acceptability and net preference

#### Mosquito and insect protection

Data from the Malaria Patients Survey (Table 1) indicated that the majority of respondents reported that mosquitoes entered the Olyset® LLINs (93%, 95CI [87.9; 96.5]), that this phenomenon occurred since the beginning or after the first wash (89.1%, 95CI [85.0; 94.1]), and that mosquitoes did not die after entering the net (89.8%, 95CI [84.5; 94.7]). In addition to their use as a malaria prevention measure, as reported in the qualitative data, users expect bed nets to safeguard them from all types of insects at night and also during the day when children and/or sick people use nets for resting. Respondents indicated that both the *virote,* the local name for “inclined” mosquitoes that are perceived to cause malaria, and the *zancudos*, the local generic name for mosquitoes, still entered the LLINs. In addition, most respondents affirmed that small insects entered the LLINs (92.4%, 95%CI [87.1; 96.0]), did so from their first days of use (85%, 95CI [78.2; 90.4]) and did not die afterwards (91.8%, 95CI [86.2; 95.7]) (Table 1). According to 93% (95%CI [87.9; 96.5]), of the respondents, traditional nets offered better protection than LLINs from mosquitoes and insects. When ranking the most important advantages and disadvantages of the LLINs versus traditional nets, poor protection against mosquitoes was the primary problem reported for LLINs (74.7% of respondents [95CI [67.2; 81.3])(data not shown).

**Table 1:** LLINs perceived protection from mosquitoes and insects.

Individual Malaria Patients Survey (N = 158)	%	n	95% CI
**Do mosquitoes enter the net (N = 158)**			
Yes	93,0	147	[87.9; 96.5]
No	4,4	7	[1.8; 8.9]
Don't know	2,5	4	[0.7; 6.4]
**Since when do mosquitoes enter the LLIN (N = 147)**			
Since the beginning	49,0	72	[40.7; 57.4]
After the first wash	40,1	59	[32.2; 48.5]
After several washes	10,2	15	[5.8; 16.3]
Missing	0,7	1	[0.0; 3.7]
**Do mosquitoes die after entering the LLIN (N = 147)**			
Yes	6,8	10	[3.3; 12.2]
No	89,8	133	[84.5; 94.7]
Don't know	2,7	4	[0.7; 6.8]
**Do small insects enter the net (N = 158)**			
Yes	92,4	146	[87.1; 96.0]
No	4,4	7	[1.8; 8.9]
Don't know	2,5	4	[0.7; 6.4]
Missing	0,6	1	[0.0; 3.5]
**Since when do small insects enter the LLIN [N = 147]**			
Since the beginning	85,0	125	[78.2; 90.4]
After the first wash	10,9	16	[6.4; 17.1]
After several washes	3,4	5	[1.1; 7.8]
Missing	0,7	1	[0.0; 3.7]
**Do small insects die after entering the LLIN (N = 147)**			
Yes	6,8	10	[3.3; 12.2]
No	91,8	135	[86.2; 95.7]
Don't know	0,7	1	[0.0; 3.7]
Missing	0,7	1	[0.0; 3.7]
**Which net offers better protection against mosquitoes/insects? (N = 158)**			
Traditional nets	93,0	147	[87.9; 96.5]
LLINs	0,6	1	[0.0; 3.5]
Both offer the same protection	3,8	6	[1.4; 8.1]
Missing	2,5	4	[0.7; 6.4]

#### Privacy requirements

Qualitative data indicated that one of the main architectural functions of the bed net was to divide the space inside the house into separate ‘rooms’ and to provide privacy. In addition, in *open houses*, one of the most important functions of the bed net was to create an inside-outside division that would otherwise be provided by walls. However, even in *closed* houses the bed net provided a degree of privacy when people rested on front porches during the day or when visitors were hosted during the night. Bed nets further established an internal division of space when various household members slept in separate beds but shared the same room. In these cases, the use of the *tocuyo* bed net helped to conceal the users, providing them with a private space, e.g. for dressing/undressing, resting and sexual activity. This was confirmed in the Malaria Patients Survey as 70.3% (95CI [62.5; 77.3]) of respondents considered the LLINs to be too transparent and 98.1% (95CI [94.6; 99.6]) stated that traditional nets ensured better privacy than LLINs (Table 2). In *closed* houses, people slept in bedrooms and bed nets were usually left hanging over the bed during the day, often to store people's clothes (Figure 1, C&D). In *open houses*, however, nets were dismantled during the day to allow the use of the same space for other purposes, such as eating or cooking. (Figure 1, A&B).

**Table 2:** Secondary benefits of bed net use (LLIN compared to *tocuyo*).

Individual Malaria Patients Survey (N = 158)	%	n	95%CI
**1. Privacy and transparency**			
**Are the LLINs:**			
Too transparent	70,3	111	[62,5; 77.3]
Transparency is OK	29,7	47	[22.7; 37.5]
**Which net is better to guard intimacy:**			
LLIN	0,6	1	[0.0; 3.5]
Traditional	98,1	155	[94.6; 99.6]
Both are the same	1,3	2	[0.2; 4.5]
**Mesh size:**			
Mesh size too big	98,7	156	[95.6; 99.8]
Mesh size is OK	0,0	0	-
Don't know	1,3	2	[0.2; 4.5]
**2. Traditional beliefs**			
**Is it a problem that one can see you inside of the LLIN?**			
Yes	46,8	74	[38.9; 54.9]
No	50,6	80	[42.6; 58.7]
Don't know	2,5	4	[0.7; 6.4]
**Is it a problem that the ** ***Tunche*** ** can see inside the net?**			
Yes	34,2	54	[26.8; 42.1]
No	61,4	97	[53.3; 69.1]
Don't know	4,4	7	[1.8; 8.9]

#### Traditional beliefs

Bed nets also played a key role in terms of traditional beliefs and practices as observed during the ethnographic phase. Bed nets were expected to conceal users from certain spirits, such as the malicious “*Tunche*”, that can harm people when sleeping. Bed nets were further required to impede their users, especially children, from seeing spirits outside such as the spirits of the dead, the sight of which can be physically and/or mentally harmful. The LLINs larger mesh size and consequent transparency were perceived to be less effective than the thicker traditional bed nets in protecting users from such spiritual forces. In the Malaria Patients Survey, almost half of respondents (46.8%, 95CI [38.9; 54.9]) expressed their concerns of being seen sleeping by other people, and about one third (34.2%, 95CI [26.8; 42.1]) expressed the same concern in relation to the exposure to the *Tunche* (Table 2).

#### Climate and seasonal net use

Users expected bed nets to provide shelter from the cold, particularly during the rainy season (often referred to as ‘the winter’), a period that corresponds with an increase in malaria transmission and when mornings are cooler. However, according to most of respondents (97.5%, 95CI [93.6; 99.3]) in the Malaria Patients Survey, LLINs did not protect against the morning chills, leading 57.6% (95CI [49.5; 65.4]) not to use them in the colder periods of the year (Table 3). Moreover, 82.9% (95CI [76.1; 88.4]) of respondents also affirmed not using LLINs during the rains. Reasons stated in the ethnographic study include both the cold weather and the perceived inefficacy of the LLINs against mosquitoes. Finally, among the 56 (35.4%) households having used at least one of the LLINs during the previous rainy season, only 23.2% (95CI [13.0; 36.4]) would use them again during the following rainy season.

**Table 3:** LLIN use in relation to seasonality and climate.

Malaria Patients Survey (n = 158)	%	n	95% CI
**LLIN too cold in the mornings:**			
Yes	97,5	154	[93.6; 99.3]
No	1,9	3	[0.4; 5.4]
Missing	0,6	1	[0.0; 3.5]
**Do you use the LLIN when it is cold?**			
Yes	38,6	61	[31.0; 46.7]
No	57,6	91	[49.5; 65.4]
Missing	3,8	6	[1.4; 8.1]
**How many LLINs did your household use last rainy season?**			
0	63,3	100	[55.3; 70.8]
≥1	35,4	56	[28.0; 43.4]
Missing	1,3	2	[0.2; 4.5]
**Do you think you will use the LLIN next rainy season (n = 56)?**			
Yes	23,2	13	[13.0; 36.4]
No	76,8	43	[63.6; 87.0]
**Do you generally use the LLIN in rainy periods?**			
Yes	17,1	27	[11.6; 23.9]
No	82,9	131	[76.1; 88.4]

#### Hygiene

The qualitative study revealed that roofs made with *irapay* (*Lepidocaryum tenue*) leaves, a local palm, attract insects and produce considerable amounts of debris falling on the beds below. Unlike the traditional *tocuyo* nets, LLINs were reported not to prevent debris and small insects from falling onto the bed, due to their large mesh size.

#### Forest activities

People engaged in economic activities in the rain forest, such as hunting and logging, stated they could only use traditional nets for sleeping in the forest. The opaque nets are reported to conceal hunters from their prey and protect against the abundance of insects.

### Housing structure and LLIN-use

Data from the case control study (Housing Study) showed that 80% of the *closed* houses used LLINs while only 40% were doing so in *open houses* (OR = 6.0; 95CI [1.8; 20.4], p = 0.0013) (Table 4). Concerning the type of net used per number of beds, 55.1% of beds were covered by LLINs in *closed* houses while this was the case in only 23.2% of beds in *open* houses (OR = 4.1, 95CI [2.0; 8.4], p<0.001). Conversely, about 35% of beds in *closed* houses were covered with traditional nets while this was the case in 70% of the *open* houses (OR = 0.22; 95CI [0.1; 0.44], p<0.001). In addition, 73.8% of the total number of LLINs received by families living in *closed* houses were actually in use while in *open* houses this was only 35.2% (OR = 5.2, 95CI [2.2; 12.3], p<0.001).

**Table 4:** Case control# study on housing and net use.

	%	n/N	OR*	95%CI	P-value
Number of households using LLIN					
Open Houses	40,0	14/35	1		
Closed Houses	80,0	28/35	6.0	[1.8; 20.4]	p = 0.0013
Kind of net used per sleeping space					
LLIN in Open Houses	23,2	19/82	1		
LLIN in Closed Houses	55,1	49/89	4.1	[2.0; 8.4]	p<0.001
Traditional net in Open Houses	70,7	58/82	1		
Traditional nets in Closed Houses	34,8	31/89	0.22	[0.1; 0.44]	p<0.001
Number of LLIN in use per total distributed:					
Open Houses	35,2	19/54	1		
Closed Houses	73,8	48/65	5.2	[2.2; 12.3]	p<0.001

# Cases were all *open houses* in the study area and Controls were their nearest neighbouring *closed houses*;

* crude OR;

After triangulating qualitative ethnographic data and the quantitative study, several factors accounting for the differences in net use between the two types of houses were identified. First, the LLINs' large mesh size and consequent transparency failed to provide the necessary privacy in *open* houses whereas in *closed* houses, walls rather than bed nets ensured this privacy. Second, LLINs did not protect from the early morning chills, which is rather a problem in *open* houses than in *closed* ones as no additional blankets are used. Third, LLINs failed to prevent debris and insects from falling into beds in houses with *Irapay* roofs, which is a more common annoyance in *open* houses that do not have the extra ceilings of *closed* houses (Table 5).

**Table 5:** Architectural/social functions of bed nets and walls by type of house.

	Open Houses	Closed Houses
1- Malaria protection	*Bed net*	*Bed net*
2- Nuisance of insects	*Bed net*	*Bed net*
3- Internal division of space	*Bed net*	Walls
4- Inside - outside division	*Bed net*	Walls
5- Privacy	*Bed net*	Walls
6- Shelter from the cold	*Bed net*	Walls
7- Increase hygiene	*Bed net*	Ceiling

### Bed net use and socio-economic status

Qualitative data clearly indicated that *open* houses usually belong to families that cannot afford to pay for the construction of walls. When followed up in the Housing Study, 91.4% of *open house* respondents would have preferred to close their homes but failed to do so due to financial constraints, in 97.1% of the cases (data not shown).

## Discussion

This study, carried out one year after mass LLIN distribution in the Amazonian region of Iquitos, identified key determinants for user preference and use. More specifically, in addition to malaria prevention, bed nets were expected to create a physical barrier providing users with privacy, sheltering them from prying eyes, to protect them from harmful spirits, to offer shelter from the morning chill during the malaria transmission and rainy season, to prevent dirt and insects from falling onto beds from *Irapay* roofs, and to protect them from the nuisance of mosquitoes and insects. However, due to the Olyset® LLINs' large mesh size and consequent transparency, they failed to meet these decisive criteria, resulting in lower use of LLINs than of traditional *tocuyo* nets. This was the case for *open* houses but also for shared and open spaces in *closed* houses. In *open* houses, the area used for domestic activities during the day was converted into a sleeping space at night by using the traditional opaque *tocuyo* nets as make-shift walls, thus ensuring their use. Even in *closed* houses certain secondary factors fostered a preference for traditional opaque nets over the newly distributed LLINs such as the need to share the same room with other household members or visitors or when sleeping outside on porches or attics under the roof.

Numerous studies have reported that secondary or complementary benefits are required to optimise bed net use [37]–[39]. Such complementary benefits often appear to be more decisive in fostering a net use culture than the originally intended benefit of malaria prevention itself [9]. In the study area, the importance of the complementary benefits was enhanced by nets' requirement to fulfil social and structural functions that in other contexts are mostly provided by architectural elements, such as inside and outside walls and ceilings. Net preference and use was therefore significantly associated with housing structure with *closed* houses being more likely to use LLINs than *open* houses. The importance of housing structure for bed net use has, similarly, been shown in Burkina Faso [40] and in Kenya [41], as net use was associated with the number of rooms per household, as well as with net availability and bed availability. Likewise, low acceptability of LLINs has been reported among other populations that require non-transparent nets for privacy [38] and among outdoor sleeping populations such as the nomadic Nuer of Southern Sudan [42]. These outdoor sleepers reported similar reasons (to the Peruvian *mestizos*) for the preference for opaque cotton bed nets, i.e. provision of shelter, privacy and protection from insects and predators. In response to this need for an outdoor LLIN, Vestergaard-Frandsen (Switzerland) designed the *Dumuria*® LLIN for nomadic populations [43].

The concern expressed on the Olyset® nets' inability to conceal users from spirits points to traditional beliefs as another impeding factor for LLIN use. Probably the survey underestimated the importance of traditional beliefs for net preference. Due to the preponderance of public health messages in the study area, we assume that a relatively high degree of social desirability bias occurred, leading to the underreporting of the newly distributed Olyset® nets' disadvantages. Nevertheless, though often underreported as not easily captured by standardized questionnaires, cultural beliefs can substantially influence or determine people's behaviour [44], with bed net use being no exception.

Our findings further showed that both use *and* preference for certain types of nets are directly related to the season. While LLINs are more accepted during the drier warmer summer months when there are fewer mosquitoes, their perceived inability to generally protect against insects and the nets' failure to shelter users from the morning chill made them unsuitable for the malaria transmission season or ‘winter’. Seasonal patterns in bed net use have also been described in contexts as varied as India [45], Sri Lanka [46] and Ghana [47]. Notably, our findings suggest that people can and do alternate between different types of bed nets depending on climatic factors and perceived mosquito density. As such, bed net use should not merely be measured as a “yes” or “no” score on one variable but as a dynamic phenomenon understood in relation to various social and environmental factors.

In relation to net users' socio-economic status, the data suggest that low-income households (those unable to afford houses with outer walls) are less likely to use LLINs than higher social classes living in closed houses. Despite the reported objections to Olyset® nets by households with *closed* houses, the nets were still used twice as often than in *open* houses. This indicates that the distribution of Olyset® LLINs were more beneficial to social classes less likely to need free-of-charge bed nets than to the poorer households. The latter had either to purchase traditional nets to compensate for their open houses' structural limitations (e.g. lack of walls and ceilings) or to use nets (LLINs) in many ways inadequate to meet their needs.

Quite unexpectedly, the distributed LLINs were perceived to be ineffective in protecting against mosquitoes and other insects. A study carried out in the Solomon Islands comparing Olyset®, Permanet® and Duranet® nets reported similar findings [48], [49]. This finding has two potentially important implications. LLINs are highly efficacious against malaria transmitting anopheles and Olyset® nets were designed with mesh small enough to prevent anopheline mosquitoes from entering the net but large enough to allow air circulation to overcome complaints of increased heat under the net [50]. Nevertheless, the perception that ‘mosquitoes’ do enter the LLIN could actually reduce their effectiveness as a malaria control tool since this leads to lower LLIN use. In addition, the complementary benefit of being protected from other insects also influences net preference and use, determining LLINs' adequacy as a malaria prevention tool. Accordingly, LLINs should be designed taking these secondary benefits/requirements into account if they are to be used against malaria in settings with a marked mosquito and insect nuisance.

Acceptability problems with ITNs not fulfilling the required social functions [22] were reported prior to the 2007 LLIN procurement in Peru, but this information was not considered in the LLINs procurement process since current policies do not allow for certain preferred net characteristics to be included into procurement decisions (i.e. mesh size, material, etc.). Focus has been placed so far on the importance of the physical durability of LLINs, which has been recognized to differ between brands of LLINs within and between countries [51]. Durability guidelines have also been produced to quantitatively measure LLIN performance in the field [52] and the WHO has published a concept note on how to improve VFM (value for money) in LLIN procurement based on the results from these studies [53]. However, without proper mechanisms measuring the impact of user preference on LLIN use and thus on malaria prevention, there will not be sufficient support for their inclusion in tenders. Social science research is essential to assess net preference and its effect on use, including cost-effectiveness studies to estimate the added value of including user preference in the procurement process on the number of effective days of LLIN protection per dollar spent. Indeed, if reducing cost and providing the greatest number of days of protection is the priority, then net preference needs to be brought into the VFM equation. Appropriate methods for studying and measuring net preference to be systematically included in procurement decisions should, therefore, be designed and prioritized.

### Limitations and strengths

The Malaria Patient Survey was not a population-based survey, and as such its results cannot be inferred to the whole population in Iquitos. Nevertheless, they apply to the population most at risk of malaria, i.e. malaria patients, representing the most interesting group in terms of targeting interventions. The Malaria Patient Survey respondents were expected to represent an unbiased sample of the population at risk of malaria since qualitative data showed that most malaria patients in the study area consult at the local health centers as these were geographically accessible (<15 km) and provided anti-malaria treatment free of charge. Furthermore, the ethnographic study did not find any differences in net preference among recently diagnosed malaria patients and the general population of the study area despite targeted research.

Secondly, the case-control study provided only crude ORs since adjustment was not possible for two reasons: i) detailed information on socio-economic status was not collected during the field study; ii) it would have been difficult to adjust for socio-economic and education status since most of the open houses belonged to the poorest families. However, the aim of the study was not to provide multivariate adjusted estimates of the effect of housing, but rather a range of the differences observed between *open* and *closed* houses. Despite these limitations, the combination of both qualitative and quantitative methods during fieldwork allowed for the confirmation of specific patterns (net preference and net use) and facilitated the detection of new and unexpected variables (such as the use of space and sleeping habits). The hypothesis that LLIN use was related to net preference and that this was the case in the most vulnerable groups for malaria was confirmed in both methodological strands.

### Conclusion

The presented research clearly shows that net preference *can* limit the optimal use of LLINs in some high-risk groups for malaria. While further studies should be carried out to confirm these results in similar settings of the Peruvian Amazon and elsewhere, net preference urgently needs to be taken into account by public health managers, donors, implementing partners, and manufacturers. In order to improve the number of effective days of LLIN protection per dollar spent, appropriate methods (quantitative and qualitative) to determine net preferences should be developed before any procurement decision is made. Malaria control policies cannot merely be based on education and behavioral change communication strategies [12], [54]. The right LLIN should reach the people most at risk of malaria and should be adequately designed for the local context.

## References

[1] Roll Back Malaria (2011) Refined/Updated GMAP Objectives, Targets, Milestones and Priorities Beyond 2011. Available: www.rbm.who.int/gmap/gmap2011update.pdf. Accessed 2011 June 9.

[2] WHO. Vector Control. Available: http://www.who.int/malaria/vector_control/en/. Accessed 2011 Dec 3.

[3] D'Alessandro U, Olaleye BO, McGuire W, Langerock P, Bennett S, et al (1995) Mortality and morbidity from malaria in Gambian children after introduction of an impregnated bednet programme. Lancet 25; 345 (8948) 479–83. 10.1016/s0140-6736(95)90582-0 7861874

[4] Lengeler C (2004) Insecticide-treated bed nets and curtains for preventing malaria (Review). Cochrane Database of Systemic Reviews 2: CD000363 doi: 10.1002/14651858.CD000363.pub2. 10.1002/14651858.CD000363.pub2 15106149

[5] Gamble CL, Ekwaru JP, ter Kuile FO (2006) Insecticide-treated nets for preventing malaria in pregnancy. Cochrane Database of Systematic Reviews (2) Art. No.: CD003755. doi: 10.1002/14651858.CD003755.pub2. 10.1002/14651858.CD003755.pub2 PMC6532581 16625591

[6] Baume CA, Marin MC (2007) Intra-household mosquito net use in Ethiopia, Ghana, Mali, Nigeria, Senegal, and Zambia: are nets being used? Who in the household uses them? Am J Trop Med Hyg 77: 963–971. 17984361

[7] Githinji S, Herbst S, Kistemann T, Noor AM (2010) Mosquito nets in a rural area of Western Kenya: ownership, use and quality. Malar J 9: 250. 20813034 10.1186/1475-2875-9-250 PMC2939624

[8] Thwing J, Hochberg N, Vanden Eng J, Issifi S, Eliades MJ, et al (2008) Insecticide-treated net ownership and usage in Niger after a nationwide integrated campaign. Trop Med Int Health 13: 827–834. 18384476 10.1111/j.1365-3156.2008.02070.x

[9] Peeters Grietens K, Xuan XN, Van Bortel W, Ngo Duc T, Ribera JM, et al (2010) Low perception of malaria risk among the Ra-glai ethnic minority in south-central Vietnam: implications for forest malaria control. Malar J 9 (23) 1–9. 20089152 10.1186/1475-2875-9-23 PMC2823606

[10] Peeters Grietens K, Nguyen Xuan X, Muela Ribera J, Ngo Duc T, van Bortel W, et al (2012) Social Determinants of Long Lasting Insecticidal Hammock-Use Among the Ra-Glai Ethnic Minority in Vietnam: Implications for Forest Malaria Control. PLoS ONE 7 (1) e29991 doi:10.1371/journal.pone.0029991. 22253852 10.1371/journal.pone.0029991 PMC3257264

[11] Pulford J, Hetzel M, Bryant M, Saba P, Mueller I (2011) Reported reasons for not using a mosquito net when one is available: a review of the published literature. Malar J 10: 83. 21477376 10.1186/1475-2875-10-83 PMC3080352

[12] Koenker H (2011) Creating a net culture. Public Health Bayer Crop Science (22) Available: http://www.bayercropscience.com/bcsweb/cropprotection.nsf/id/EN_Public_Health_Journal/file/PHJ_22.pdf. Accessed 2012 Dec 5

[13] WHO (2011) Inception meeting for the pilot-study project on sustainable management of long-lasting insecticidal nets throughout their life-cycle. Available: http://www.who.int/malaria/publications/atoz/who_htm_gmp_2011_1/en/index.html. Accessed 2011 Oct 12.

[14] WHOPES (2011) Long Lasting Insecticidal Mosquito Nets. Available: http://www.who.int/whopes/Long_lasting_insecticidal_nets_Jul_2012.pdf. Accessed 2012 Jan 10.

[15] R4D (2012) Expanding Access to LLIN: a Global Market Dynamics Approach. Available: http://www.resultsfordevelopment.org/sites/resultsfordevelopment.org/files/R4D_LLIN%20report_24Apr_Final.pdf. Accessed 2012 Oct 18.

[16] Global Fund to Fight Aids, Tuberculosis and Malaria (2011) Country Coordinating Mechanisms. Available: http://www.theglobalfund.org/en/ccm/guidelines. Accessed 2011 Dec 3.

[17] Quick Facts on Procuring Long-lasting Insecticidal Nets. The Global Fund Aids Tuberculosis and Malaria. Available: http://www.theglobalfund.org/en/procurement/quality/. Accessed 2011 June 9.

[18] Alliance for Malaria Prevention Toolkit 2.0 (2011) Chapter 4: LLIN procurement and pipeline monitoring. Available: http://www.allianceformalariaprevention.com/resources-view.php?categoryID=29. Accessed 2011 Sept 6.

[19] Roper M, Carrion Torres R, Cava Goicochea C, Anderson E, Aramburu Guarda J, et al (2000) The Epidemiology of Malaria in an Epidemic Area of the Peruvian Amazon. Am J Trop Med Hyg 62 (2) 247–256. 10813480 10.4269/ajtmh.2000.62.247

[20] Llanos-Zavalaga F, Huayta Zacarias E, Lecca García L (2005) Factores asociados al uso de mosquiteros en el departamento de Piura, Perú. Rev Med Hered 16: 97–106.

[21] Harvey SA, Paredes Olórtegui M, Winch PJ, Leontsini E, Gilman RH (2000) Insecticide-impregnated mosquito nest for malaria control in the Iquitos region of the Peruvian Amazon— Formative Research for an Efficacy Trial. November 1999–July 2000: report on social, cultural, and economic factors (unpublished).

[22] Harvey SA, Paredes Olórtegui M, Leontsini E, Bustamante Pezo C, Olórtegui Pezantes LM, et al (2008) The Whole World Will Be Able to See Us: Determining the Characteristics of a Culturally Appropriate Bed Net Among Mestizo Communities of the Peruvian Amazon. Am J Trop Med Hyg 79 (6) 834–838. 19052288

[23] Organismo Andino de Salud-Convenio Hipólito Unanue. Proyecto PAMAFRO. Available: http://www.orasconhu.org. Accessed: 2006 Nov 13.

[24] Rosas-Aguirre Angel, Guzmán-Guzmán Mitchel, Gutierrez Diamantina Moreno, Rodriguez -Ferrucci Hugo, Vargas-Pacherrez Daniel, et al (2011) Posesión, Retención y Uso de Mosquiteros Tratados con Insecticidas de Larga Duración Luego de un Año de su Distribución en Loreto, Perú. Rev Peru Med Exp Salud Publica 28 (2) 228–36. 21845302 10.1590/s1726-46342011000200009

[25] OGEP (2006) Oficina General de Epidemiología. Peru. Weekly Epidemiological Bulletin. Lima: OGEP.

[26] Peeters Grietens K, Soto V, Erhart A, Muela Ribera J, Toomer E, et al (2010) Adherence to 7-Day Primaquine Treatment for *P.vivax* Radical Cure in the Peruvian Amazon. Am J Trop Med Hyg 82 (6) 1017–1023. 20519594 10.4269/ajtmh.2010.09-0521 PMC2877405

[27] Van den Eede P, Soto-Calle VE, Delgado C, Gamboa D, Grande T, et al (2011) Plasmodium vivax Sub-Patent Infections after Radical Treatment Are Common in Peruvian Patients: Results of a 1-Year Prospective Cohort Study. PLoS ONE 6 (1) e16257 doi:10.1371/journal.pone.0016257. 21297986 10.1371/journal.pone.0016257 PMC3030575

[28] Oficina General de Epidemiologia, DIRESA Loreto (2008) *Análisis de la situación de salud de la region Loreto año 2007.* Available: http://www.dge.gob.pe/pub_asis. Accessed 2012 Feb 10.

[29] Aramburu Guarda J, Ramal Asayag C, Witzig R (1999) Malaria Reemergence in the Peruvian Amazon Region. Emerging Infectious Diseases 5 (2) 209–215. 10221872 10.3201/eid0502.990204 PMC2640690

[30] Schoeler G, Flores-Mendoza C, Fernandez R, Davila J, Zyzak M (2003) Geographical Distribution of *Anopheles Darlingi* in The Amazon Basin Region of Peru. Journal of the American Mosquito Control Association 19 (4) 286–296. 14710728

[31] Vittor A, Gilman R, Tielsch J, Glass G, Shields T, et al (2006) The Effect of Deforestation of the Human-Biting Rate of *Anopheles Darlingi*, the Primary Vector of Falciparum Malaria in the Peruvian Amazon. Am J Trop Med Hyg 74 (1) 3–11. 16407338

[32] Mendis K, Sina B, Marchesini P, Carter R (2001) The Neglected Burden of *Plasmodium Vivax* Malaria. Am. J Trop Med Hyg 64 (1,2): S: 97–106. 10.4269/ajtmh.2001.64.97 11425182

[33] PAMAFRO (2011) Reporte de Resultados. Available: http://www.orasconhu.org/sites/default/files/1ReporteResultadosNo10-JUL2010PAMAFRO_1.pdf. Accessed 2012 Feb 10.

[34] Teddlie C, Tashakkori A (2008) Foundations of Mixed Methods Research: Integrating Quantitative and Qualitative Approaches in the Social and Behavioral Sciences. London: Sage.

[35] American Anthropological Association (1998) Code of Ethics of the American Anthropological Association. Available: www.aaanet.org/committees/ethics/ethcode.htm. Accessed 2008 Sept 14.

[36] American Anthropological Association (2004) American Anthropological Association Statement on Ethnography and Institutional Review Boards. Available: http://www.aaanet.org/stmts/irb.htm. Accessed 2008 Sept 14.

[37] Sexton J (1990) Permethrin-Impregnated Curtains and Bed-Nets Prevent Malaria in Western Kenya. Am Soc Trop Med Hyg 43: 11–18. 10.4269/ajtmh.1990.43.11 2200287

[38] Winch PJ, Makemba AM, Kamazima SR, Lwihula GK, Lubega P, et al (1994) Seasonal Variation in the Perceived Risk of Malaria: Implications for the promotion of insecticide-treated nets. Soc Sci Med 39: 63–75. 8066488 10.1016/0277-9536(94)90166-x

[39] Aikins MK, Pickering H, Greenwood BM (1994) Attitudes to malaria, traditional practices and bednets (mosquito nets) as vector control measures: a comparative study in five West African countries. J Trop Med Hyg 97: 81–86. 8170007

[40] Toe LP, Skovmand O, Dabire KR, Diabate A, Diallo Y, et al (2009) Decreased motivation in the use of insecticide-treated nets in a malaria endemic area in Burkina Faso. Malar J 8: 175. 19640290 10.1186/1475-2875-8-175 PMC2729312

[41] Iwashita H, Dida G, Futami K, Sonye G, Kaneko S, et al (2010) Sleeping arrangement and house structure affect bed net use in villages along Lake Victoria. Malaria J 9: 176. 10.1186/1475-2875-9-176 PMC2906499 20569459

[42] Bean J (2001) Insecticide Treated Bed Net Trials, South Sudan. Roll Back Malaria/WHO, ECHO. (Unpublished)

[43] RBM (2001) Final Report Insecticide Treated Bed Nets Trials: Lankien, Bieh State Upper Nile, South Sudan. Available: http://www.rbm.who.int/partnership/country/docs/EAfrica/reaping_sudan.pdf. Accessed 2012 Feb 10.

[44] Helman C (2007) Culture, Health and Illness. London: Hodder Arnold.

[45] Gunasekaran K, Sahu SS, Vijayakumar KN, Jambulingam P (2009) Acceptability, willing to purchase and use of long lasting insecticide treated mosquito nets in Orissa State, India. Acta Trop 112 (2) 149–55. 19631186 10.1016/j.actatropica.2009.07.013

[46] Fernando SD, Abeyasinghe RR, Galappaththy GN, Gunawardena N, Rajapakse LC (2008) Community factors affecting long-lasting impregnated mosquito net use for malaria control in Sri Lanka. Trans R Soc Trop Med Hyg 22: 222–223. 10.1016/j.trstmh.2008.06.007 18644611

[47] Agypeong I, Manderson L (1999) Malaria Prevention in the Greater Accra Region, Ghana: mosquito avoidance and bed net use. J Biosocial Sci 31: 79–92. 10.1017/s0021932099000796 10081239

[48] Atkinson JA, Bobogare A, Vallely A, Boaz L, Kelly G, et al (2009) A cluster randomized controlled cross-over bed net acceptability and preference trial in Solomon Islands: community participation in shaping policy for malaria elimination. Malar J 16;8 (1) 298. 10.1186/1475-2875-8-298 PMC2803192 20015402

[49] Atkinson JA, Bobogare A, Fitzgerald L, Boaz L, Appleyard B, et al (2009) A qualitative study on the acceptability and preference of three types of long-lasting insecticide-treated bed nets in Solomon Islands: implications for malaria elimination. Malar J 4;8: 119. 10.1186/1475-2875-8-119 PMC2699345 19497127

[50] Olyset Net Technical Information (2011) Sumitomo Chemical. Available: http://www.olyset.net/olysetnet/. Accessed 2012 Feb 10.

[51] WHO (2009) Report of the twelfth WHOPES working group meeting. Geneva: World Health Organization. Available: http://whqlibdoc.who.int/hq/2009/who_HTM_NTD_whoPES_2009_1_eng.pdf. Accessed 2012 Feb 10.

[52] WHO (2011) Guidelines for monitoring the durability of long lasting insecticidal mosquito nets under operational conditions. Available: http://whqlibdoc.who.int/publications/2011/9789241501705_eng.pdf. Accessed: 2012 Feb 10.

[53] WHO (2011) A system to improve Value for Money in LLIN procurement through market competition based on cost per year of effective coverage: Concept Note. Available: http://www.who.int/malaria/publications/atoz/gmpllin_effective_coverage_concept_note/en/index.html. Accessed 2012 Feb 10.

[54] Brieger W (1994) Monitoring community response to malaria control using insecticide-impregnated bed nets, curtains and residual spray at Nsukka, Nigeria. Health Education Research 11: 133–145. 10.1093/her/11.2.133-a 10163407

